# Emergence of social behavior deficit, blunted corticolimbic activity and adult depression-like behavior in a rodent model of maternal maltreatment

**DOI:** 10.1038/tp.2016.205

**Published:** 2016-10-25

**Authors:** M Rincón-Cortés, R M Sullivan

**Affiliations:** 1 Department of Child and Adolescent Psychiatry, Child Study Center, New York University Langone Medical Center, New York, NY, USA; 2 Neuroscience and Physiology, Sackler Institute for Graduate Biomedical Studies, New York University School of Medicine, New York, NY, USA; 3 Emotional Brain Institute, Nathan Kline Institute for Psychiatric Research, Orangeburg, NY, USA

## Abstract

Disrupted social behavior is a core symptom of multiple psychiatric and neurodevelopmental disorders. Many of these disorders are exacerbated by adverse infant experiences, including maltreatment and abuse, which negatively affect amygdala development. Although a link between impaired social behavior, abnormal amygdala function and depressive-like behavior following early adversity has been demonstrated in humans and animal models, the developmental emergence of maltreatment-related social deficits and associated amygdala neural activity are unknown. We used a naturalistic rodent model of maternal maltreatment during a sensitive period, postnatal days 8–12 (PN8–12), which produces social behavior deficits that precede adolescent depressive-like behavior and amygdala dysfunction, to examine social behavior in infancy, periweaning and adolescence. Neural activity in response to the social behavior test was assessed via c-Fos immunohistochemistry at these ages. A separate group of animals was tested for adult depressive-like behavior in the forced swim test. Maltreatment spared infant (PN16–18) social behavior but disrupted periweaning (PN20–22) and adolescent (PN42–48) social behavior. Maltreated rats exhibited blunted neural activation in the amygdala and other areas implicated in social functioning, including the medial prefrontal cortex and nucleus accumbens, at these ages and increased adult depressive-like behavior. These findings may suggest corticolimbic involvement in the emergence of maltreatment-induced social deficits that are linked to adult depressive-like behavior, thereby highlighting potential targets for therapeutic intervention. Understanding how infant experiences influence social behavior and age-specific expression across development may provide insights into basic neural mechanisms of social behaviors and disease-relevant social dysfunction exacerbated by early-life stress.

## Introduction

Social behavior deficits are a hallmark feature of psychiatric and neurodevelopmental disorders, including depression, anxiety, autism and schizophrenia,^1, 2, 3, 4^ and are associated with abnormal amygdala structure and function.^5, 6, 7, 8^ Animal models suggest a causal link between social deficits and the amygdala.^9^ Amygdala involvement in social behavior has been demonstrated by (i) lesion studies in rodents^10^ and nonhuman primates,^11, 12^ (ii) socially evoked changes in neuronal firing activity within the basolateral amygdala (BLA),^13^ (iii) bidirectional modulation of social behavior via optogenetic manipulation of BLA fibers^14, 15^ and (iv) neuroimaging studies of social cognition in humans.^16^ Here we extend this work to include social behavior deficits within an animal model of depressive-like behavior induced by early-life experience with a maltreating mother.

Importantly, many psychiatric and neurodevelopmental disorders have origins in early life and are exacerbated by stressful infant experiences including early-life abuse and maltreatment, which alter the brain development and increase the risk for later-life psychopathologies such as depression.^17, 18, 19, 20, 21, 22^ Adverse early-life experiences involving the caregiver negatively affect the development of the amygdala^23, 24^ a critical brain area for emotion and social behavior^25, 26^ in humans^27, 28, 29, 30, 31^ and other mammals.^24, 32, 33, 34, 35^ Furthermore, maltreatment and abuse lead to social impairments,^36, 37, 38, 39^ which typically precede the onset of later-life psychopathology and serve as a predictive marker for later-life symptoms related to psychopathology.^40, 41^ Similar findings have been obtained using naturalistic rodent models of early-life stress that mimic maternal maltreatment.^42^

The amygdala has a key role in social behavior,^25^ the long-term effects of childhood abuse/maltreatment^17, 21, 27^ and the pathophysiology of depression,^43, 44^ a common outcome of early-life abuse.^18, 20, 43, 44, 45^ A link between deficient social behavior, abnormal amygdala function and depressive-like behavior following early-life adversity has been demonstrated in humans^46, 47^ and rodent models.^42, 46, 47, 48^ Despite these findings, the developmental emergence of social behavior deficits and related neural activity following maltreatment are unknown. To this end, we used a rodent model of maternal maltreatment that consists of creating a low resource environment (that is, insufficient bedding for nest building) for the dam from postnatal (PN) days 8–12, which stresses the mother and increases the frequency of negative maternal behaviors that are painful to the pups, although pups maintain normal weight gain.^34, 49^ This model closely reflects clinical literature indicating that abused and/or maltreated children exhibit social behavior dysfunction and are at increased risk for developing later-life depression,^37, 40, 41^ as we have previously shown that maltreatment-induced social deficits serve as a predictive marker for adolescent depressive-like behavior and amygdala dysfunction.^42^ Our lab has characterized PN8–12 as a sensitive period in the amygdala development during which alterations in the maternal behavior owing to low-bedding stress result in long-lasting social behavior deficits and later-life depressive-like behavior mediated by the amygdala.^22, 23, 24, 42^ However, the ontogeny of social behavior deficits, as well as brain regions associated with disrupted social behavior, has not been explored.

To this end, we assessed the emergence of maltreatment-induced social behavior deficits and associated neural activity patterns through immunohistochemical detection of c-Fos protein expression across early development (that is, infancy, periweaning, adolescence) in rats. Neural activity in response to the social behavior test was examined within the amygdala as well as in other brain areas sensitive to early-life stress and implicated in the neurobiology of social behavior and depression,^4, 50, 51, 52, 53, 54^ such as the medial prefrontal cortex (mPFC)^55, 56, 57, 58^ and the nucleus accumbens (NA).^53, 59, 60^ We focused on the lateral, basal and central amygdala because previous work suggests that these subnuclei are selectively affected by maltreatment at the ages explored here (that is, periweaning, adolescence).^42^ Briefly, the lateral amygdala is the major site receiving inputs from sensory systems and generally viewed as the gatekeeper: the basal amygdala receives inputs from the lateral amygdala and connects with the central amygdala as well as other striatal areas involved in the control of instrumental behaviors, while the central nucleus is an important output region for the expression of emotional responses and associated physiological responses.^26, 61^ Finally, since childhood maltreatment/abuse is a risk factor for adult depression in humans,^17, 18, 45^ we tested adult depressive-like behavior in the forced swim test (FST)—a measure of behavioral despair in rodents.^62^

## Materials and methods

### Animals

Male and female Long–Evans rats born and bred in our colony were housed in polypropylene cages (34 × 29 × 17 cm) with an abundant amount of wood shavings for nest building, and kept in a 20±1 °C environment with a 12:12 light–dark cycle. Food and water were available *ad libitum*. The day of birth was considered PN0, litters were culled to 12 pups (six males, six females) on PN1 and the rats were weaned on PN23. To avoid possible confounding of litter effects with variables of interest, no more than one male and female animal from a given litter was assigned to an experimental condition and at least four different litters per infant condition (ran over separate cohorts) were used in all the experimental procedures. The sample sizes were chosen on the basis of previously reported findings.^42^ All animal care and experimental procedures were approved by the Institutional Animal Care and Use Committee, which follow the guidelines from the National Institute of Health.

### Rodent model of maternal maltreatment

The mother and her pups were housed in a cage with limited (100 ml) nesting/bedding material (that is, alpine shavings, Northeastern Product, Warrensburg, NY, USA) from PN8 to PN12. This bedding manipulation alters the maternal behavior and increases negative behaviors painful to the pup such as stepping, dragging and rough handling, which involves improper transport of pups (that is, picking it up and moving it (anywhere but the nest)) as well as the frequency of audible pup vocalizations (Supplementary Table 1).^24, 34, 42, 48, 49^ Notably, this procedure mimics the effects of a stressful rearing environment (that is, resource depletion) as a risk factor for potentiating infant abuse.^24, 49^ This paradigm is similar to the more stressful low-bedding manipulation developed in the Baram laboratory,^63^ where more stressors (that is, grid floor, unchanged bedding) and significant reduced pup weight gain could model much greater adversity.^63, 64^ Control mothers and pups were housed in a cage with abundant nesting/bedding material (~4500 ml) from PN8 to PN12, which allows the mother to build an adequate nest and spend most of her time caring for pups.^42, 48, 63, 64^

### Behavioral studies

#### Social behavior test

The social approach behavior was tested as previously described,^42^ during infancy (PN16–18; *n*=7 control, *n*=5 maltreated), periweaning (PN20–22; *n*=6 control, *n*=6 maltreated) and adolescence (PN42–48; *n*=7 control, *n*=7 maltreated). The animals from both infant conditions were tested and scored blind on the same day. Briefly, each animal received a 5-min acclimation period in the testing apparatus. After habituation, the rat was removed from the testing apparatus and a younger same sex (that is, social stimulus) animal was placed inside one of a metal cube, which allows for olfactory, auditory and tactile communication but prevents aggressive or sexual interactions.^65^ The test animal was placed in the control chamber and the number of chamber crossings and time spent in the social stimulus chamber was recorded and scored for 10 min. Social behavior was measured as the total time spent in the social stimulus chamber, as previously reported by our laboratory^42, 48, 66^ and total number of chamber crossings was used as an index of general locomotor activity.^42^ Decreased time spent in the social compartment compared with the non-social compartment is defined as social avoidance and thought to reflect a reduction in social motivation.^67, 68^

#### Forced swim test

The FST is a measure of behavioral despair in which rodents are forced to swim under inescapable conditions and the duration of immobility behavior is recorded.^66, 69^ Rats (*n*=7 control, *n*=7 maltreated) were tested for depressive-like behavior in the FST during adulthood (>PN75) using a transparent acrylic cylinder (36.8 × 36.8 × 47 cm) filled with clean water (25±1 °C; depth prevented escape and tail touching bottom) for each animal and without knowledge of the experimental condition. The animals underwent two swim sessions on two consecutive days. Day 1 consisted of a 15-min pretest swim to habituate the rats to the test situation, thereby providing a stable, high level of immobility during the 5-min test on the following day (day 2).^62, 70^ Two parameters of depressive-like behavior were recorded and scored blindly: time spent immobile, defined as passive floating without struggling, slightly hunched but upright position with minor movements to maintain head above water,^42, 48, 70^ as well as the latency to immobility—the first time at which the animal initiated a stationary posture that did not reflect attempts to escape/struggle. This passive posture had to last 5 s or longer to be scored as an immobility bout. The rats were gently dried, placed on a heated chamber and returned to the home cage after both sessions.

### Neural assessment

We used c-Fos protein expression as a metabolic marker of cell activation.^71^ Although resting-state levels of c-Fos are typically low, physiological or psychosocial challenges induce the expression of c-Fos protein, which serves as an indirect marker for neuronal activity.^71, 72^ The animals were decapitated 90 minutes following the end of the social behavior test because peak expression of c-Fos occurs around this time.^71, 72^ The brains were removed, frozen and stored in a −80 °C freezer until sectioning in a Leica CM3050S cryostat (20 μm) at −20 °C. The brains were cut in two series: every fourth section was collected for c-Fos immunohistochemistry and the next section was collected for cresyl violet staining so that the distance between each fos-stained section is 80 μm. The sections received a 15 min post-fix in 4% paraformaldehyde/0.1 m phosphate-buffered saline (PBS, pH 7.4). Following fixation, the sections were rinsed in PBS three times. To eliminate peroxidase activity, the sections were incubated in 3% H_2_O_2_ and 97% methanol for 15 min. Following four PBS rinses, the slides (Fisherbrand, Fisher Scientific, Pittsburgh, PA, USA) were incubated in a blocking solution containing 1% normal goat serum (Jackson ImmunoResearch Laboratories, West Grove, PA, USA; Catalog No. 005-000-121) and 1% albumin for 30 min. The slides were then treated overnight at room temperature with the primary antibody (anti-c-Fos (Ab5) (4-17) Rabbit pAb, Calbiochem, San Diego, CA, USA; Product No. PC38-100UL) diluted 1:1000 in blocking solution. Afterwards, they were rinsed in three PBS washes and incubated in the secondary antibody (goat anti-rabbit IgG, Vector Labs, Burlingame, CA, USA; Catalog No. BA-1000) diluted 1:200 in 50% blocking solution for 30 min at room temperature followed by additional PBS rinses. The sections were treated for 30 min in avidin–biotin–peroxidase complex solution (ABC Elite kit, Catalog No. PK-6101, Vector Labs) and the slides were then rinsed three times in PBS and treated with a solution containing Vector VIP (VIP), H_2_O_2_ and nickel (Vector VIP peroxidase kit, Catalog No SK-4600; Vector Labs) for 5 min, rinsed in PBS, subsequently dehydrated in alcohol and xylene, and coverslipped for microscope examination.

The c-Fos-positive cells were counted bilaterally and the brain areas were outlined using a stereotaxic rat brain atlas.^73^ All the c-Fos-positive cells were distinguished from the background by density of staining, shape and size of cells and were counted without knowledge of the experimental condition. The mean bilateral count of the number of cells containing c-Fos for an animal was determined by averaging the counts from three sections per brain area, as described previously.^34^ The brain areas examined included the basal and lateral amygdala nuclei, the PFC cingulate, prelimbic (PL) and infralimbic (IL) cortices, and the NA core and shell.

### Statistical analysis

The social behavior/chamber crossing data were analyzed with two-way analysis of variance followed by *post hoc* Fisher tests. The c-Fos data and adult FST data were analyzed by *t*-tests. The data were expressed as mean (±s.e.m.) and in all the cases, differences were considered significant when *P*<0.05.

## Results

### Maternal maltreatment disrupts social behavior during periweaning and adolescence, but not during infancy

**Figure 1:**
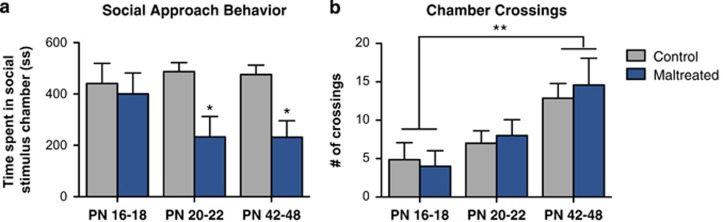
Developmental emergence of social behavior deficits following maternal maltreatment. (**a**) Maltreated rats show normal social behavior during infancy (that is, postnatal day (PN)16–18). Periweaning rats (that is, PN20–22) and adolescent (that is, PN42–48) experiencing maternal maltreatment exhibit reduced social approach behavior compared with controls. (**b**) Maltreated animals do not differ from control animals in the number of chamber crossings at any age, although adolescent rats of both groups (control, maltreated) exhibited a higher amount of chamber crossings; *P<0.05, ** *P*<0.01. Error bars represent s.e.m. (*n*=5–7 per group).

The exposure to caregiver maltreatment from PN8 to PN12 spared infant social behavior but impaired periweaning and adolescent sociability (Figure 1), confirming a developmental delay for the emergence of social behavior deficits as the pups approach weaning.^42^ Although infant (that is, PN16–18) rats reared with an maltreating mother exhibited social behavior that did not differ from controls, periweaning (that is, PN20–22) and adolescent rats exposed to infant maltreatment spent significantly less time in the social chamber than rats reared with a normal mother (F_(1,31)_=9.996, *P*<0.05; Figure 1a), which is thought to reflect social avoidance.^68^ Significant effects of age were found for chamber crossings (F_(2,32)_=12.27, *P*<0.01; Figure 1b). Maltreated animals did not differ from control animals in the number of chamber crossings at any age, although adolescent (PN42–48) rats of both groups (control, maltreated) exhibited a higher amount of chamber crossings than infant (PN16–18) rats. These findings suggest that although there is an age-related increase in locomotor activity, the observed effects of maltreatment at periweaning and adolescent are not owing to between-group differences in locomotion.

### Developmental emergence of social behavior deficit following maternal maltreatment is associated with blunted cellular activation within corticolimbic structures

#### Amygdala

**Figure 2:**
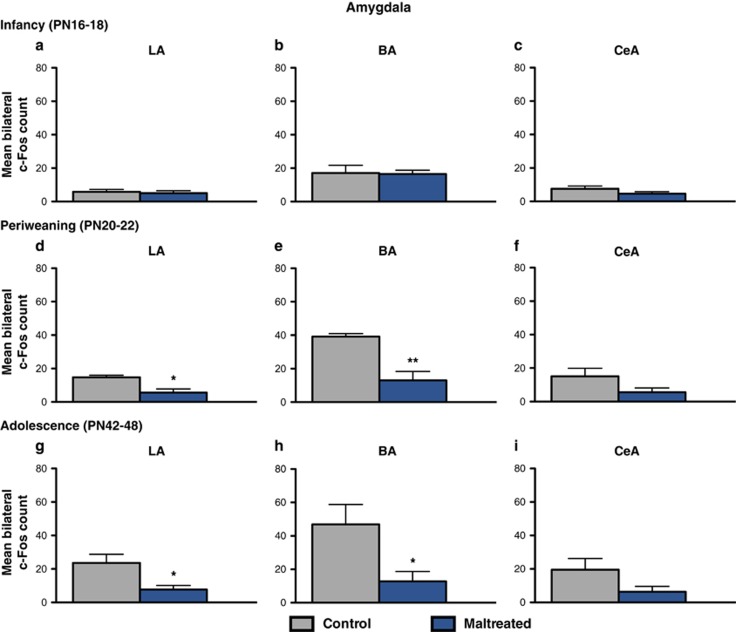
Amygdala neural activity in response to the social behavior test at infancy, periweaning and adolescence. (**a–c**) At postnatal day (PN)16–18, maltreated rats showed no significant difference in c-Fos expression in the lateral, basal or central amygdala nuclei compared with control animals. (**d**–**f**) Periweaning (PN20–22) and adolescent (PN42–48) rats exposed to maltreatment exhibited a significant reduction in c-Fos expression in the lateral (**d** and **g**) and basal (**e** and **h**) amygdala nuclei in response to the social behavior test compared with control animals (*n*=4–6 per group; *P*<0.05); *P<0.05, **P<0.01. Bars represent the number (mean±s.e.m.) of c-Fos positive cells counted bilaterally in the lateral and basal amygdala nuclei. BA, basal amygdala; CeA, central amygdala; LA, lateral amygdala.

Infant maltreatment attenuated amygdala activation within the lateral and basal amygdala nuclei in response to social behavior testing during periweaning and adolescence, but not during infancy (Figure 2). Periweaning animals reared with a maltreating mother from PN8 to 12 exhibited a reduction of c-Fos positive cells in the lateral (*t*=3.318, df=7; *P*<0.05, Cohen's *d*=2.324, effect size *r*=0.758) and basal (*t*=4.197, df=7; *P*<0.01, Cohen's *d*=2.981, effect size *r*=0.830) amygdala nuclei compared with controls (Figures 2d and e). No difference was found between the control and maltreated rats in the central amygdala nuclei (*P*=0.1074; Figure 2f). A similar pattern was found in maltreated adolescent animals, which also had lower counts of c-Fos positive cells in the lateral (*t*=2.795, df=10; *P*<0.05, Cohen's *d*=1.613, effect size *r*=0.628) and basal (*t*=2.555, df=10; *P*<0.05, Cohen's *d*=1.475, effect size *r*=0.594) amygdala nuclei, but not the central (*P*=0.1053) compared with control animals reared by a normal mother (Figures 2g–i).

#### Prefrontal cortex

**Figure 3:**
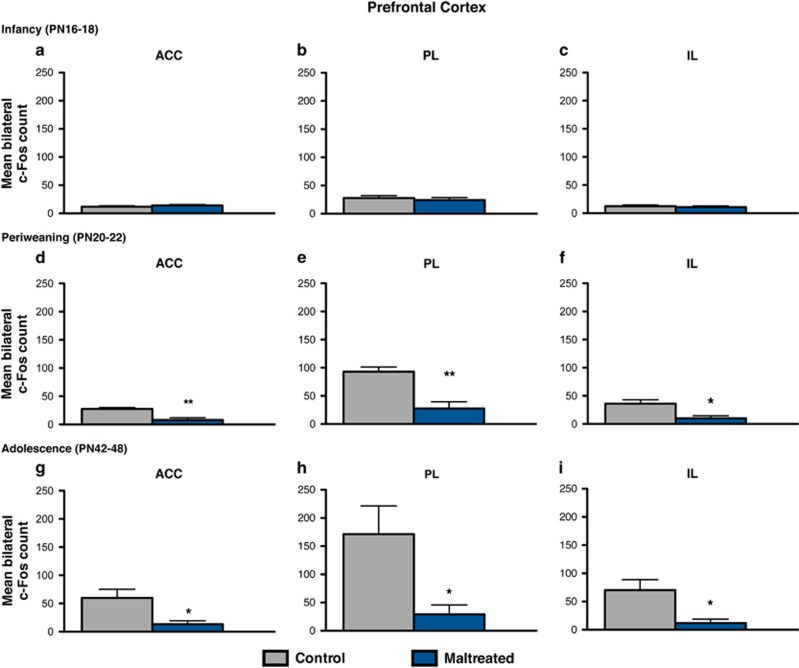
Maltreatment effects on cellular activation in response to social behavior testing within the mPFC during infancy, periweaning and adolescence. (**a**–**c**) At postnatal day (PN)16–18, no significant differences were found in any of the medial prefrontal cortices in response to the social behavior test between maltreated and control animals. (**d**–**f**) Periweaning animals that were maltreated from PN8 to PN12 exhibited decreased cellular activation in the mPFC, as indicated by lower counts of c-Fos expression, in the cingulate (**d**), prelimbic (**e**) and infralimbic (**f**) cortices compared with control animals (*n*=4–5 per group; *P*<0.05). (**g**–**i**) A similar pattern was observed in maltreated adolescent animals, which also showed attenuated c-Fos expression in the cingulate (**g**), prelimbic (**h**) and infralimbic (**i**) cortices compared with control animals (*n*=5–6 per group; *P*<0.05); *P<0.05; **P<0.01. Bars represent the number (mean±s.e.m.) of c-Fos positive cells counted bilaterally in each nuclei. ACC, anterior cingulate cortex; IL, infralimbic cortex; mPFC, medial prefrontal cortex; PL, prelimbic cortex.

Maternal maltreatment dampened c-Fos protein expression in the mPFC of periweaning and adolescent animals, but not infant animals (Figure 3). The assessment of neural activity in the mPFC, including the anterior cingulate (ACC), PL and IL cortices, in response to the social behavior test revealed a widespread reduction of c-Fos immunoreactivity in each of these subdivisions during the periweaning (Figures 3d–f) and adolescent (Figures 3g–i) periods following early-life abuse. Periweaning animals exposed to maltreatment exhibited decreased c-Fos expression in the ACC (*t*=3.375, df=7; *P*<0.01, Cohen's *d*=2.613, effect size *r*=0.794), PL (*t*=4.216, df=7; *P*<0.01, Cohen's *d*=2.916, effect size *r*=0.825) and IL (*t*=3.342, df=7; *P*<0.05, Cohen's *d*=2.189, effect size *r*=0.738) compared with control animals (Figures 3d–f). Adolescent animals experiencing infant maltreatment also showed a similar reduction in the ACC (*t*=2.610, df=9; *P*<0.05, Cohen's *d*=1.648, effect size *r*=0.636), PL (*t*=2.483, df=9; *P*<0.05, Cohen's *d*=1.571, effect size *r*=0.618) and IL (*t*=2.731, df=9; *P*<0.05, Cohen's *d*=1.723, effect size *r*=0.653) compared with control animals (Figures 3g–i).

#### Nucleus accumbens

**Figure 4:**
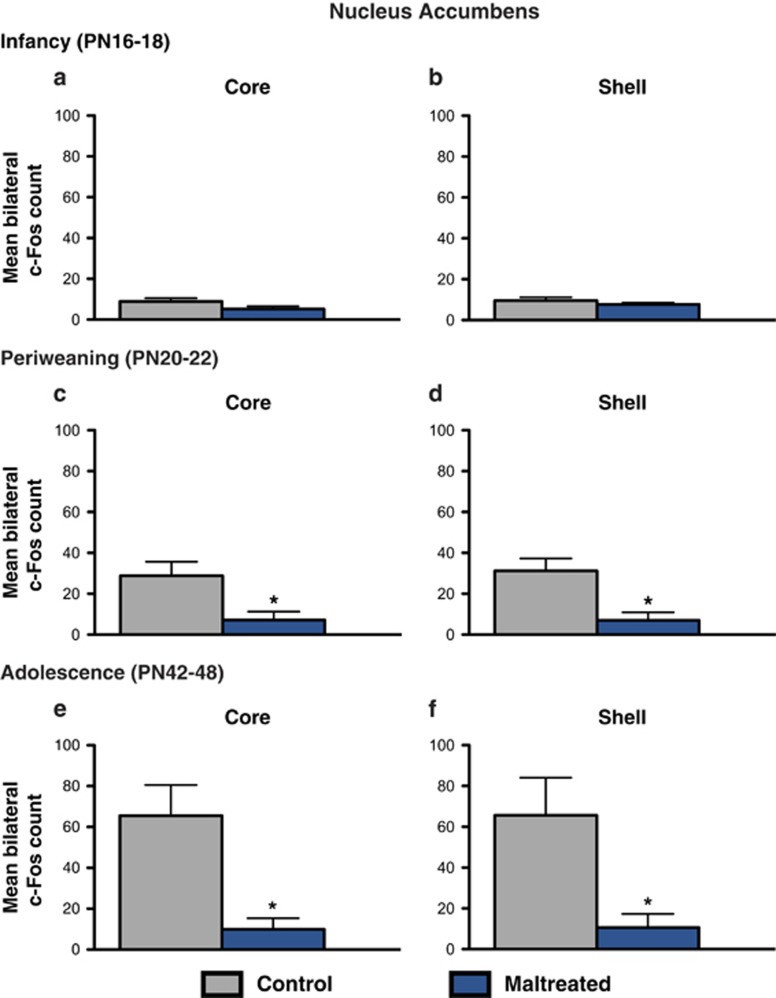
Activity in the nucleus accumbens in response to the social behavior test across development. (**a** and **b**) During infancy (postnatal day (PN)16–18), no differences were found in the NA core or shell of maltreated and control animals. (**c** and **d**) Periweaning (PN20–22) animals exposed to maternal maltreatment had reduced c-Fos expression in both the NA core and shell compared with control animals (*n*=4 per group; *P*<0.05). (**e** and **f**) Previously maltreated adolescent animals also had reduced activation (that is, lower number of c-Fos positive cells) in the NA core and shell (*n*=5–6 per group; *P*<0.05). *P<0.05. Bars represent the number (mean±s.e.m.) of c-Fos positive cells counted bilaterally in each nuclei. NA, nucleus accumbens.

Similar to the amygdala and the mPFC, maternal maltreatment resulted in blunted c-Fos expression following social behavior testing that was specific to the periweaning and adolescent periods (Figure 4). Periweaning animals receiving infant maltreatment had diminished c-Fos counts in both the NA core (*t*=2.656, df=6; *P*<0.05, Cohen's *d*=1.878, effect size *r*=0.685) and NA shell (*t*=3.322, df=6; *P*<0.05, Cohen's *d*=2.350, effect size *r*=0.761) compared with control animals (Figures 4c and d). This decline in the number of c-Fos immunoreactive cells was also observable in the NA core (*t*=3.202, df=9; *P*<0.05, Cohen's *d*=2.024, effect size *r*=0.711) and shell (*t*=2.589, df=9; *P*<0.05, Cohen's *d*=1.636, effect size *r*=0.633) of adolescent animals experiencing maternal maltreatment (Figures 4e and f).

### Maternal maltreatment increases immobility duration and decreases latency to immobility in the FST during adulthood

**Figure 5:**
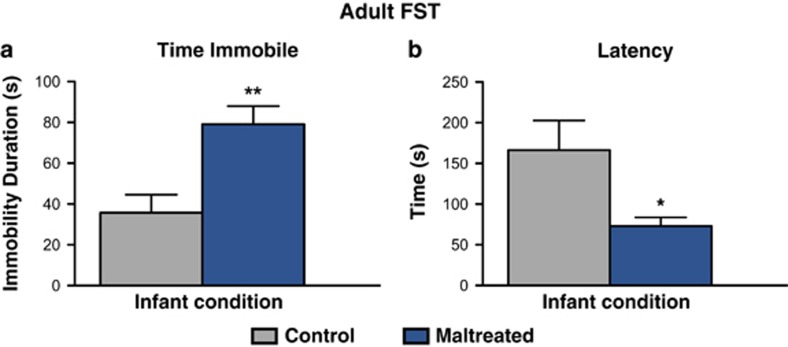
Maternal maltreatment programs adult depressive-like behavior in the FST. (**a**) Maltreated adult rats displayed increased immobility duration (*n*=7 per group; *P*<0.01) in the FST compared with control animals reared with a normal mother from postnatal day (PN)8 to PN12. (**b**) Maltreated animals also exhibited a reduced latency to immobility (*n*=7 per group; *P*<0.05) compared with controls; *P<0.05, **P<0.01. Error bars represent s.e.m. FST, forced swim test.

Infant maltreatment induced adult depressive-like behavior in the FST across two parameters: time spent immobile and latency to immobility (Figure 5). Maltreated rats reared displayed increased immobility duration (that is, passive floating) during the FST compared with controls (*n*=7 per group; *t*=3.462, df=12; *P*<0.01, Cohen's *d*=1.850, effect size *r*=0.679; variance, *P*=0.9851; Figure 5a), which was accompanied by a reduction in the latency to immobility (*n*=7 per group; *t*=2.468, df=12; *P*<0.05, Cohen's *d*=1.319, effect size *r*=0.550; variance, *P*=0.0080) compared with controls (Figure 5b). Collectively, these findings suggest that early life abuse, as modeled by maternal maltreatment, increases behavioral despair in response to the adult FST.

## Discussion

Adverse social experiences during early life are associated with marked dysfunction in social functioning across the lifetime.^67^ Here we demonstrate that maternal maltreatment, as modeled by rearing PN8–12 pups with dam provided with insufficient bedding for nest building, induced long-lasting changes in sociability that were characterized by a decrease in social approach behavior (that is, social avoidance; Figure 1), thought to reflect a reduction in social motivation.^67, 68^ These data corroborate prior findings showing that early-life caregiver maltreatment results in atypical social behavior during periweaning (PN20–22) and adolescence (PN42–47; Figure 1),^42^ but broadens these results to younger ages and expands on brain areas closely associated with affect and social behavior (Figures 2, 3, 4).^50, 69, 74^ The inclusion of a younger age group (that is, PN16–18) indicates that social behavior deficits emerge later in development, as pups approach independence, and are accompanied by neural alterations in the amygdala, NA and PFC (Figures 2, 3, 4)—all of which are part of the social motivation network in humans and rodents.^4, 25, 50, 67^ Recent work from our laboratory has shown that aberrant social behavior following infant maltreatment persists into adulthood,^48^ which is consistent with preclinical reports that adult sociability is disrupted by prenatal, neonatal and juvenile stress exposure, all of which reduce social motivation and inhibit social interactions.^67^ Furthermore, these findings are in accordance with clinical studies indicating that childhood maltreatment is linked to impaired social skills^46, 47^ and is a strong predictor of later-life social behavior problems, including adolescent social withdrawal and adult antisocial behavior.^37, 38, 75^

Identification of neural substrates implicated in the developmental disruption of social behavior by caregiver maltreatment is an important translational goal, as it may provide insight into potential therapeutic targets for correcting social behavior dysfunction present in depression and other affective disorders exacerbated by early-life adversity. Here we examined the long-term effects of early-life stress, as modeled by caregiver maltreatment, on neural activation in a subset of brain regions implicated in the social brain, which refers to brain areas activated in humans in social cognition tasks,^73^ following social behavior testing. Notably, the social brain network in other mammals overlaps with the human social brain, including the amygdala, prefrontal cortex and the ventral striatum.^25, 50, 67, 73^ Infant maltreatment dramatically dampened neural activation in these corticolimbic structures in response to the social behavior test, as indicated by a widespread reduction of c-Fos immunoreactivity in the BLA, mPFC and NA (Figures 2, 3, 4). Moreover, these effects were specific to infant condition and age and were only observable in relation to social behavior deficits. These findings are in agreement with previous reports demonstrating blunted neural activity within these structures in adolescent rats subjected to postweaning social isolation stress. Previous studies have identified changes in c-Fos expression in rats younger than those included in this study (that is, <PN15), suggesting that the lack of neural activity changes during infancy (that is, PN16–18) is not due to a methodological floor effect.

Maternal maltreatment attenuated amygdala activity in the lateral and basal nuclei, but not the central nuclei, in response to the social behavior test at periweaning and adolescence (Figure 2). This is consistent with preclinical findings of dampened socioemotional behavior and reduced basolateral amygdala neuronal excitability following prenatal stress^78^ and with clinical reports of dysfunctional social approach behavior in depressed patients, which is correlated with a strong decrease in amygdala activation.^7^ The amygdala's role in social behavior is complex, as it has extensive connections to other subcortical and cortical structures whose function it modulates.^25, 50, 79^ For example, the amygdala connects with prefrontal and striatal areas implicated in guiding social affiliation or avoidance, such as the mPFC and NA.^80, 81, 82^ Indeed, social play behavior in rats increases neural activity (that is, enhances c-Fos immunoreactivity) in the amygdala, mPFC and NA, and correlations between social play behavior and cellular activation in cortico–amygdala and amygdalo–striatal connections have been suggested.^83^

Social interactions, including social approach behavior and social play, activate neurons in the mPFC,^83, 84^ and pharmacological inactivation of the mPFC reduces social interaction.^85^ Consistent with these data, infant maltreatment reduced social behavior (Figure 1) and blunted neural activity in the PFCs (that is, cingulate, PL, IL) at both periweaning and adolescence (Figure 3). This is of clinical relevance because adults with a history of childhood maltreatment exhibit hypoactive mPFC function.^57^ Moreover, the mPFC, which shares reciprocal connections with the BLA,^86, 87^ exhibits profound alterations in a variety of neurodevelopmental and psychiatric disorders involving impaired social cognition and dysregulated affect, including depression.^52, 88^ Furthermore, early-life adversity such as childhood maltreatment and/or maternal deprivation alters amygdala–PFC connectivity^56^ and results in structural abnormalities in both structures.^29, 58^ Such changes are also frequently found in individuals with abnormal social behavior.^67^ In rodents, the BLA–mPFC pathway has a causal role in the bidirectional modulation of social behavior.^14^

A similar decline in neural activation in response to social behavior testing was also observed in the NA core and shell (Figure 4), which receive projections from both the mPFC and the amygdala that are involved in the motivational aspects of behavior.^80, 81, 89^ Although the NA had previously been implicated in social play behavior,^83^ a causal role for the NA in social approach/interaction behavior has recently been revealed. In female rats, increases in the activity of ventral tegmental area dopamine neurons and the ventral tegmental area–NA pathway encode and predict key features of social behavior through a dopamine D1-receptor mechanism.^90^ The NA has also been implicated in both the pursuit of social reward and the avoidance of social punishment in humans.^91^

The alterations in the connections between the amygdala, the mPFC and the NA have been implicated in social inhibition,^92, 93^ depression^7, 94^ and the neurobiological sequelae of early-life stress.^33, 53, 56^ For example, functional changes in the mPFC can cause prominent changes in social behavior in both humans and other mammals owing to its projections to subcortical limbic structures involved in initiating behaviors related to the motivational significance of sensory stimuli, like the amygdala and the NA.^89, 95^ In humans, high levels of social inhibition—the tendency to withdraw from new people and avoid social situations—are associated with reduced connectivity within limbic, striatal and prefrontal regions.^92^ In rodents, early social stressors, postweaning social isolation and chronic adult stressors reduce activation in most areas of the social brain when animals are exposed to other conspecifics^76, 77, 78, 96^ (Figures 2, 3, 4), which is consistent with general impairment of social behaviors induced by such stressors.

Intriguingly, many brain areas particularly vulnerable to early-life stress, such as the amygdala, mPFC, NA, are characterized by protracted postnatal development, high density of glucocorticoid receptors and exhibit functional and/or structural alterations in individuals with abnormal social behavior.^21, 24, 54, 97^ Collectively, these findings suggest that the developmental trajectory of these structures is sensitive to early-life adversity, which programs later-life social behavior deficits by altering the way cortical and limbic structures respond to social encounters, which may enhance susceptibility towards developing additional symptoms relating to psychopathology. However, it is important to note that the effects of early-life stress are ubiquitous throughout the brain and there are additional areas not examined here, such as the hippocampus and hypothalamic areas, that may be affected by maltreatment and contribute to the social dysfunction and increased risk for psychopathology associated with early adversity.^73, 98^

As we integrate these findings into previous work from our lab, it becomes clear that infant maltreatment produces task-specific changes in brain activity patterns. For example, maltreated adolescents have attenuated amygdala responses in the social behavior test but exhibit a hyperactive response to an inescapable, uncontrollable stressor (that is, FST) during the same developmental period.^42^ Generalized statements about early-life experiences attenuating or potentiating neural activity should include task-specific information, which likely use different circuits within a brain area. Furthermore, the amygdala's contribution to social behavior is not rigid and universal,^4^ but context-dependent and susceptible to individual differences.^50^ Effects of early adversity on social behavior changes and associated neural structures may reflect a change in the way context-dependent situations (stimuli in the context of an emotionally significant or socially significant setting) modulate motivated behavior. Given that the social experience (that is, time spent in social chamber) was lower for maltreated animals, an alternative explanation may be that the behavioral differences between each group induced reduced social stimuli exposure, which induced the Fos difference. In this case, our Fos results may reflect differences in stimulus exposure owing to individual-initiated activity differences in these areas after maternal maltreatment, which may lead to a change in their developmental trajectories. Indeed, in the human literature, reduced social interaction is thought to initiate a developmental cascade that can potentiate the effects of early-life adversity.^36, 99, 100^ However, even when stimulus exposure is controlled, previous work from our lab suggests neural differences between maltreated and control animals. Specifically, weaning-aged pups show significant differences in amygdala and PFC in response to other social odors (maternal and adult male odors) even when exposure time is controlled in weaning-aged pups.^101^ Furthermore, maltreated animals without stimulus exposure exhibit alterations in resting-state functional connectivity between the ACC/mPFC and amygdala, as well as between the ACC/mPFC and the striatum compared with control animals, and some of these connectivity patterns change from adolescence to early adulthood.^102^ Together, it is likely that adversity-induced brain changes are further modified by behavior differences induced by the adversity.

In addition to social behavior deficits and neural alterations, maltreatment induced adult depressive-like behavior, as indexed by increased immobility duration and reduced latency to immobility in the FST (Figure 5). We have previously shown that depressive-like behavior in the FST emerges during adolescence and is associated with enhanced amygdala activity in the lateral, basal and central amygdala nuclei.^42^ Amygdala hyperactivity is causal in the expression of depressive-like behavior, as pharmacological inactivation of the amygdala via muscimol infusion before the FST rescues depressive-like behavior.^42^ Thus, our rodent model of early-life abuse recapitulates findings in humans indicating that early stress stemming from childhood adversity is a predisposing risk factor for the development of social deficits and adult depression.^103^

In summary, here we used a naturalistic rodent model of chronic early-life stress to demonstrate long-term effects of early-life adversity on social behavior. This paradigm recapitulates the neurobehavioral sequelae of abused children, including social behavior deficits that are frequently comorbid with depression and usually precede the expression of depression-related symptoms.^46, 47^ This is exemplified by abuse-induced social behavior deficits that emerge at periweaning and persevere throughout the lifetime,^42, 48^ although depressive-like behavior in the FST emerges in adolescence^42^ and persists into adulthood. This alteration in social behavior is associated with blunted activation of corticolimbic regions that comprise the social brain, including the BLA, the mPFC and the NA, and are critically involved in the neurobiology of mood and psychiatric disorders, including depression.^54, 88, 97^ Thus, these data support human and preclinical research indicating that adverse early-life events involving the caregiver negatively affect the developmental trajectory and function of cortical and limbic regions implicated in decision-making, emotion and social behavior.^23, 31, 33, 56^ Finally, our findings provide insight into the mechanisms by which early-life stress affects brain structures implicated in learning, reward processing, motivation and sociability^51^ and the involvement of these areas in the onset of disease-relevant social dysfunction exacerbated by early-life stress.
