# Supplementary material for: Emergence of social behavior deficit, blunted corticolimbic activity and adult depression-like behavior in a rodent model of maternal maltreatment
Source: Transl Psychiatry. 2016 Oct 25;6(10):e930–. doi: 10.1038/tp.2016.205 (PMC5290349; doi:10.1038/tp.2016.205)
Supplement: Supplementary Table 1 [file tp2016205x1.pdf]

Table 1. Negative maternal and pup behaviors observed during the low bedding (i.e. maltreatment) procedure.

	Control Mother	Maltreating Mother
**Maternal behaviors**		
Step on pups	0.00%	15.28%
Drag pups	1.39%	9.72%
Rough handling	1.39%	13.19%
**Pups vocalization**	0.00%	16.67%

Values are the percentage of observation periods in which behaviors occurred. Maternal behaviors were recorded (5-minute segments; 30 minute sessions) from PN8-12 during different times of the day (AM/PM). Litters were not disturbed while being observed.
